# Engaging Nurses in Effective Cost of Care Conversations to Address Cancer-Related Financial Toxicity: Results from an Exploratory Survey

**DOI:** 10.3390/curroncol32010033

**Published:** 2025-01-08

**Authors:** Jean S. Edward, Amanda Thaxton Wiggins, Louis G. Baser, Haafsah Fariduddin, Joanna F. Doran, Monica F. Bryant, John A. D’Orazio, Kimberly D. Northrip

**Affiliations:** 1 College of Nursing, University of Kentucky, Lexington, KY 40536, USAlouisbaser3@uky.edu (L.G.B.); 2 Markey Cancer Center, University of Kentucky, Lexington, KY 40536, USA; haafsah.fariduddin@uky.edu (H.F.);; 3 Triage Cancer^®^, Chicago, IL 60646, USA; jd@triagecancer.org (J.F.D.); mb@triagecancer.org (M.F.B.); 4 Department of Pediatrics, College of Medicine, University of Kentucky, Lexington, KY 40508, USA

**Keywords:** financial toxicity, cost of care conversations, nurse training, financial and legal navigation

## Abstract

Few evidence-based trainings exist on how to equip healthcare providers, particularly nurses, with the skills to engage in cost of care conversations with patients/caregivers to mitigate the impact of cancer-related financial toxicity. This study evaluated a pilot training developed in collaboration with Triage Cancer^®^ to prepare oncology nurses to identify and assist patients/caregivers facing financial and/or legal barriers to care. Ten pediatric oncology nurses completed the training and pre/post-surveys on behaviors related to financial and legal need screening, frequency and comfort level of answering questions, knowledge, and behavior changes, along with training evaluation questions. At baseline, six nurses reported never screening for financial needs and nine for legal needs. Following the training, seven nurses stated they were likely to screen for financial/legal needs. At six months post-training, nurses had referred 85 patients/caregivers to financial/legal navigation services. Comfort levels in answering financial/legal questions increased by 6.5 points and knowledge scores increased by 1.7 points post-training. Most nurses recommended this training to other healthcare providers who work with patients with cancer and their caregivers. This study highlights the importance of providing oncology nurses with resources to engage in cost of care conversations and oncology financial legal navigation programs to mitigate the impact of cancer-related financial toxicity.

## 1. Introduction

As the prevalence and incidence of cancers in the United States increase, so does the cost of cancer care, estimated to total USD 246 billion in national expenditures by 2030 [1,2,3]. These increased costs raise the risk for patients experiencing cancer-related financial toxicity (FT), which encompasses the psychological, behavioral, and material hardships associated with paying for cancer treatment [4,5,6]. Addressing FT at the point of care is essential and requires a holistic, multidisciplinary approach to appropriately engage in cost of care (CoC) conversations, which involves physicians, nurse practitioners, nurses, social workers, and financial navigators, among others. Cost of care conversations involve the early screening and identification of patients and caregivers experiencing health-harming social, financial, and legal barriers to care and timely referrals to financial and legal navigation resources.

There is a paucity of information on how oncologists can mitigate the impact of FT on health outcomes by engaging in CoC conversations with patients/caregivers, [7,8], with even fewer discussions centered around the role of clinical oncology nurses. Oncologists express hesitancy with engaging in CoC conversations with patients due to their limited time, knowledge, skills, and referral resources [9]. However, when trained, oncologists demonstrate increased knowledge related to financial needs, financial resources, and how to navigate CoC discussions [10]. Currently, no such training programs to engage nurses in CoC conversations to address cancer-related financial toxicity exist. Like oncologists, a bedside nurse may not have the skills, time, and capacity to address all aspects of managing costs of patient care. However, having the baseline knowledge to be able to identify unmet financial and legal needs to proactively help patients and their caregivers obtain access to resources is an essential and central part of their role [11]. As one of the most trusted healthcare professionals with a focus on patient advocacy, nurses are strongly positioned to lead CoC conversations with patients and caregivers to connect them with timely and appropriate FT resources [12].

In our prior work, we implemented *FINassist-Train* (*Financial and Insurance Navigation Assistance-Training*), an interdisciplinary training program designed to promote CoC conversations and address cancer-related FT [11]. The program demonstrated improvements in knowledge of financial and legal resources, confidence in engaging in CoC conversations, and ability to address social and financial needs among healthcare providers. However, our training was not geared towards clinical oncology nurses, and few programs exists that specifically train nurses on how to engage in CoC conversations to address FT. In response to this gap, we developed a similar training for oncology nurses in collaboration with Triage Cancer^®^. Triage Cancer^®^ is a national non-profit organization providing education and navigation services to help address financial, legal, and practical issues experienced by patients with cancer, caregivers, and healthcare staff [13]. The training consisted of a 4 h self-paced online course and 4 h in-person training based on Triage Cancer’s^®^ resources [13] and topics related to social determinants of health, financial and legal barriers to care, and resources to address these barriers. In this study, we piloted the training and evaluated its impact on knowledge, behaviors, perceived impact, and attitudes related to screening for financial/legal needs, engaging in CoC conversations, and referring patients to financial/legal navigation services among nurses in one oncology clinical setting.

## 2. Methods

This study was approved by the University of Kentucky’s Institutional Review Board (#85255) and is a part of a larger project implementing and evaluating a virtual Oncology Financial and Legal Navigation (OFLN) intervention within an oncology clinic affiliated with a National Cancer Institute designated comprehensive cancer center. This clinic serves pediatric, adolescent and young adult patients with cancer. The training program described in this study was designed to prepare clinic nurses to engage in CoC conversations to better screen for and identify patients and caregivers with unmet financial and legal needs who would benefit from participating in the larger OFLN intervention. Nurses were engaged in referring and consenting patients/caregivers for participation in the OFLN intervention.

Triage Cancer^®^ helped adapt their existing 8 h financial and legal intensive workshop for healthcare professionals and advocates (which is offered for free to all who register) into a 4 h self-paced online course and a 4 h in-person training. The 4 h online course included educational recorded webinars, short, animated videos, and materials on the following topics: health insurance, including Medicaid, employment rights, disability insurance, wage replacement options for caregivers, financial management, financial assistance programs that address social determinants of health, and other legal and practical issues that may impact patients and caregivers. The online course also included an overview of the services provided by Triage Cancer^®^, including the Legal & Financial Navigation Program. Following the completion of the online course, nurses completed a 4 h in-person, comprehensive training consisting of in-depth information on health and disability insurance, employment rights of caregivers, strategies for managing finances, and other valuable information on how nurses can help identify financial and legal barriers to care and help mitigate the financial impact of a cancer diagnosis across the continuum of care. The in-person training included a review of key topics from the online course as well as interactive case studies to allow attendees to apply their knowledge.

We used a pre–post-survey design guided by the Kirkpatrick–Barr Education Evaluation Framework to evaluate the training across four levels (reaction, learning, behavior, and results). Clinic nurses who accepted the invitation to participate in the training completed consents and baseline surveys prior to completing the training. Nurses completed the self-paced, online training between November 2023 and January 2024 and participated in the in-person training in February 2024. Nurses completed post-training surveys following the in-person training. They received USD 150 for completing the surveys and 8 h of training (4 h online course and 4 h in-person training).

Outcome measures were collected across the Kirkpatrick–Barr Education Evaluation Framework’s four levels of training evaluation including *reaction*, *learning*, *behavior*, and *results*. The baseline/pre-training survey captured demographics such as race, ethnicity, age, and years practicing as a nurse. It also measured screening behaviors (*behavior*) related to financial and legal needs (e.g., in the last 6 months, how many times have you screened, referred, and/or provided direct assistance for patients/caregivers for legal or financial needs). The pre- and post-training surveys collected data on the frequency of requests for guidance (i.e., not at all, somewhat frequently, frequently, and very frequently) from patients/caregivers on 11 topics including health insurance options, appealing health insurance claim denials, helping navigate short- and long-term disability insurance options, appealing disability insurance claim denials, managing finances, medical bills, costs of care, financial assistance, food stamps, transportation, and supplemental income. It also asked comfort level (*learning*) of nurses in providing guidance (i.e., not comfortable, somewhat comfortable, comfortable, and very comfortable) on the above 11 topics. For both frequency and comfort 11-item scales, summative scores were calculated at each time point, yielding a potential range of 11–44 for each. Both surveys also included six knowledge-based questions (*learning*) on health insurance, Medicare, medical bills, Family and Medical Leave Act (FMLA), reasonable accommodations for work, and Social Security Disability Insurance (SSDI). A total knowledge score was calculated for both time points, as the total number of six responses participants answered correctly. The post-survey captured likelihood for behavior change (*behavior*) related to financial and legal screening, referring, and assisting as well as training evaluation questions (*reaction*). Actual referrals 6 months following training was also captured (*results*). An open-ended question was also asked to capture feedback beyond the Likert scales.

Frequency distributions were used to summarize nurse demographic variables and financial and legal behaviors. Means and standard deviations for frequency and comfort total scores were presented, as well as frequency distributions for each item, with the last two response options combined for analysis purposes. Means and standard deviations for total knowledge scores for pre- and post-assessment were presented, as well as frequency distributions for each knowledge item. Wilcoxon signed-rank tests were used to evaluate changes in frequency, comfort, and knowledge total scores. Training evaluation questions were also summarized descriptively using means and standard deviations. All analyses were conducted using SAS, version 9.4, with an alpha level of 0.05.

## 3. Results

A total of 10 nurses participated in this training. Most identified their race as White and non-Hispanic (n = 9), and the majority were between 20 and 40 years of age (n = 6). Nursing experience was well distributed among this sample, with three nurses having 1–5 years, three with 6–10 years, one with 11–20 years, and three with 21 or more years of nursing experience.

Financial behaviors: Most nurses reported never having screened a patient and/or caregiver for financial needs (n = 6), and half of them had never provided direct assistance with accessing resources that help patients and/or caregivers with financial needs. However, all reported having referred a patient and/or caregiver to someone who can provide financial navigation assistance, with five nurses referring 1–5 patients, two referring 6–10 patients, and three referring over 10 patient referrals.

Legal behaviors: Screening, providing resources, and referring patients and/or caregivers with respect to legal needs were much less frequently reported. Only one in ten nurses reported having screened for legal needs and having provided direct assistance with accessing resources that help patients and/or caregivers with legal needs. The majority of nurses had never referred a patient and/or caregiver to someone who can provide legal navigation assistance.

Frequency of patients asking for guidance: There was a slight, yet non-significant, increase from pre-training to post-in-person assessment for frequency of patients and/or caregivers asking for guidance (see Table 1). Based on a potential range of 11–44, although mean scores increased from 14.0 (SD = 2.9) on the pre-assessment to 15.8 (SD = 2.3) on the post-assessment, they remained relatively low (*p* = 0.078). On the pre-assessment, with the exception of transportation, nearly all nurses reported infrequent experiences with patients and/or caregivers asking them about health insurance options, appealing general and disability claim denials and financial assistance. On the post-assessment, there was a slight increase in the frequency of patients and/or caregivers asking about all items, with the most notable increases related to health insurance options, medical bills, and financial assistance.

Comfort answering questions: Nurses reported significantly higher comfort in answering patient and/or caregiver questions from the pre- to post-assessment (Table 1). In the pre-assessment, the average comfort scores were close to the potential minimum score of the scale (*M* = 12.0; SD = 0.9) and increased to an average of 18.5 (SD = 5.7; *p* = 0.006), but were still relatively low (potential range: 11–44). Like the frequency items, with the exception of transportation, most nurses reported little comfort with regard to answering patient and/or caregiver questions for all items, and the most notable increases in comfort post-training were reported for health insurance options, medical bills, and financial assistance.

Knowledge: Based on a total of 6 knowledge items, there was a nearly significant increase in the number of correct responses from 2.2 in the pre-assessment (SD = 1.4) to 3.9 in the post-assessment (SD = 1.2; *p* = 0.051; see Table 2). From the pre- to post-assessment, there was an increase in knowledge on when patients should pay their medical bill (50% correct responses in the pre-assessment versus 100% correct in the post-assessment; see Table 2), SSDI and long-term disability benefits, and knowledge related to reasonable accommodations. However, nurses were still unsure about what is included in the out-of-pocket maximum and Medicare Part B coverage.

Behavior change: When asked about changes in their behavior as a result of completing the training, the majority of nurses said they were somewhat likely or very likely to screen patients for financial or legal needs (n = 7) and provide direct assistance with accessing resources (n = 8); all respondents said they were likely to refer patients/caregivers to someone who can provide financial or legal navigation assistance. At six months following training, nurses had referred a total of 85 patients and caregivers to receive financial and/or legal navigation assistance.

Thoughts on training: With a potential range of 0 ‘not at all’ to 4 ‘very much’, nurses had the highest agreement with the statement regarding recommending the training to other healthcare providers who work with patients/caregivers with childhood, adolescent and young adult cancers (*M* = 3.1; SD = 0.7; see Table 3). The next highest rated items were being more willing to use the information resources and training to engage in assisting with patients’ legal or financial needs (*M* = 2.9; SD = 0.9) and the training improving their ability to assist with patient/caregiver financial and/or legal needs (*M* = 2.9; SD = 0.7).

## 4. Discussion

In this study, we piloted and evaluated the impact of a training that was developed in partnership with Triage Cancer^®^ to prepare oncology nurses to identify and assist patients/caregivers facing financial and legal barriers to care. We found significant pre/post-training changes in comfort answering patient/caregiver questions and knowledge. Prior to completing the training, the majority of nurses had never screened for financial or legal needs. However, as a result of completing the training, seven nurses indicated a high likelihood of screening patients/caregivers for financial or legal needs. At six months post-training, 85 patients and caregivers had been screened and referred to the OFLN services. These pre/post-training trends correspond with a 6.5-point increase in nurse comfort level of answering financial and legal questions. Lastly, nurses supported the idea of the training being offered to other healthcare professions that work with patients with cancer and their caregivers. These results demonstrate that, when trained, nurses could confidently and accurately screen for financial and legal needs among their patients, thus providing a more holistic approach to addressing cancer-related financial toxicity.

Prior to the training, nurses indicated that they were less likely to screen, assist, and refer patients with cancer and their caregivers to financial and legal resources. The current literature demonstrates that providers believe that screening for social determinants is needed; however, there are a lack of resources and trainings that make clinicians feel comfortable and confident in their ability to do so [14,15]. A key barrier for nurses is the lack of training on identifying and intervening on the social determinants of health within nursing curricula [16]. While efforts are being taken to increase nursing education around screening for social determinants of health [17], there is limited discussion around the role of nurses in engaging in CoC conversations specifically on legal barriers to care. In this study, only one nurse screened for legal needs in comparison to four nurses that screened for financial needs. It is important for nurses to engage in screening for health-harming financial and legal barriers to care because they are uniquely positioned to identify and refer patients/caregivers to limited readily available resources [11,17,18]. This is applicable to healthcare systems that employ other healthcare professionals (s.a. social workers, financial navigators, etc.) to address these issues and in settings that do not. By engaging nurses in CoC conversations, we are adding another layer of screening and an additional touchpoint to ensure there are no missed opportunities to connect patients/caregivers with resources. The training described in this study addresses key gaps in the nursing literature by equipping oncology nurses with the resources and skills necessary to properly screen for, assist, and refer patients/caregivers to financial and legal resources.

Nurses reported that few patients/caregivers asked them questions about financial and legal barriers to care, and nurses had correspondingly low levels of comfort in answering those questions. The most frequently asked questions were related to financial assistance and costs of care; however, the majority of nurses indicated being ‘not at all’ comfortable answering financial assistance and cost-of-care questions. The low frequency of patients/caregivers directing questions to nurses could be related to the corresponding low levels of health and insurance literacy in this population [19]. Individuals with low health literacy are noted to be more passive within healthcare settings, limiting the quality of interactions, despite experiencing higher unmet social needs related to chronic conditions like cancer [20]. Essentially, patients do not know what they do not know, which places a responsibility on healthcare providers to help initiate CoC conversations. Studies show that oncologists do not feel comfortable answering financial questions due to a lack of education and training [7,8,9]. Fewer studies focus on engaging providers, specifically clinical oncology nurses, on discussing health-harming financial and legal needs with patients [11]. As a result of completing our training, nurses reported higher comfort levels with answering financial and legal questions in each subcategory, thereby illustrating the potential of oncology nurses to engage in effective CoC conversations.

Nurses in our study demonstrated low health insurance literacy, with an average of 2.2 out of 6 knowledge questions answered correctly at baseline and a 3.9 average post-training. Most notably, the lowest pre-training scores were found for questions on Medicare Part B and SSDI, and the lowest post-training scores were found for the out-of-pocket maximum question. This corresponds with existing studies demonstrating low levels of health insurance literacy in the general U.S. population [19]. Additional research is needed to examine health insurance literacy needs of nurses and other healthcare providers and understand that it is associated with the ability to engage in effective CoC conversations. With further education and training, nurses’ knowledge and comfort level in helping patients/caregivers navigate financial and legal barriers to care could improve.

Overall, our pilot training had a positive impact on the comfort, knowledge, and ability/likelihood of our sample of oncology nurses to screen for financial and legal needs of patients and caregivers. A high referral rate of 85 patients and caregivers at six months post-training reflects the direct impact of the training on nurse behaviors related to engaging in effective CoC conversations to refer patients/caregivers to OFLN resources. Nurses strongly recommended this training to other nurses, healthcare providers, and staff who work with patients with cancer and their caregivers. Similarly, our previous *FINassist-Train* intervention [10], which demonstrated improvements in knowledge and confidence among oncologists, was also rated highly for being logical, appropriate, and effective in helping them engage in effective CoC conversations and ability to address social and financial needs among oncologists. As this training employed evidence-based and readily available Triage Cancer^®^ resources, it can be easily adapted for delivery in other healthcare settings.

While the training and resources are widely relevant, scalable, and provided for free via Triage Cancer^®^, it is important to consider costs and other resources associated with program implementation. The program’s core components, such as understanding health insurance options, managing healthcare finances, and addressing the social determinants of health, are universally applicable in different healthcare contexts and align with the principles outlined in Future of Nursing 2020–2030 Report by the National Academy of Medicine [17]. These foundational training components address common financial health challenges associated with social determinants of health faced by patients and caregivers across various settings. Among other trainings, Triage Cancer^®^ offers their 8 h healthcare professional training (which was adapted in this study) for free along with readily available online resources and educational materials to help navigate cancer-related financial and legal issues. With support from research funding, Triage Cancer^®^ adapted their 8 h training into a 4 h online and a 4 h in-person trainings to accommodate staffing schedules and preferences. Furthermore, training materials were tailored to meet the needs of our pediatric oncology patient and caregiver population. While there may be upfront costs associated with preparation and provision of adapted training materials and potential opportunity costs of nurses helping patients navigate financial/legal needs, the training’s scalability and adaptability may help offset these over time. By ensuring scalability and contextual needs, this training program has the potential to influence how nurses engage in CoC conversations to help identify and intervene in financial and legal barriers to care.

There are several limitations in our study that limit generalizability, and thus, a cautious interpretation of the study findings is required. This training was tested in a specific clinic to prepare a small sample of nurses to help screen, recruit, and consent patients and caregivers in an OFLN intervention. Educational resources were specifically curated to match the needs of the pediatric and adolescent and young adult oncology population and nursing staff needs. The clinic also has a robust psychosocial team with social workers and a financial counselor who help address specific social and financial needs, which could influence levels of engagement among nurses. Despite these limitations, our study indicates that this training and other evidence-based Triage Cancer^®^ resources geared towards healthcare professionals could be readily delivered in healthcare settings to facilitate effective patient–provider CoC conversations.

Our study findings highlight the importance of engaging oncology nurses in CoC conversations and OFLN to potentially mitigate the impact of cancer-related financial toxicity on patients and caregivers. Educating nurses on how to identify unmet financial and legal needs could provide them with the tools necessary for proactive screening and referral to timely and appropriate OFLN resources. Further research is needed to test the impact of this training in a larger sample of nurses across different settings and to effectively engage nurses in interdisciplinary OFLN programs.

## Figures and Tables

**Table 1:** Frequency of asking and comfort with answering patient and/or caregiver questions pre- and post-training (*N* = 10).

	Frequency of Patients and/or Caregivers Asking for Guidance		Comfort Answering Questions	
	Pre	Post	Pre	Post
Total score (potential range 11–44), *mean* (SD)	14.0 (2.9)	15.8 (2.3)	12.0 (0.9)	18.5 (5.7)
Health insurance options, n				
Not at all	10	7	10	2
Somewhat	0	3	0	5
Frequently/comfortable or very frequently/comfortable	0	0	0	3
Appealing health insurance claim denials, n				
Not at all	9	8	10	5
Somewhat	1	2	0	4
Frequently/comfortable or very frequently/comfortable	0	0	0	1
Help navigating short- and long-term disability insurance options, n				
Not at all	9	9	10	5
Somewhat	1	1	0	5
Frequently/comfortable or very frequently/comfortable	0	0	0	0
Appealing disability insurance claim denials, n				
Not at all	10	9	10	5
Somewhat	0	1	0	5
Frequently/comfortable or very frequently/comfortable	0	0	0	0
Managing their finances, n				
Not at all	9	9	10	5
Somewhat	1	1	0	4
Frequently/comfortable or very frequently/comfortable	0	0	0	1
Medical bills, n				
Not at all	7	4	10	4
Somewhat	3	6	0	4
Frequently/comfortable or very frequently/comfortable	0	0	0	2
Cost of care, n				
Not at all	8	5	10	6
Somewhat	2	5	0	2
Frequently/comfortable or very frequently/comfortable	0	0	0	2
Financial assistance, n				
Not at all	6	3	8	3
Somewhat	3	6	2	5
Frequently/comfortable or very frequently/comfortable	1	1	0	2
Food stamps, n				
Not at all	8	6	8	6
Somewhat	1	2	2	4
Frequently/comfortable or very frequently/comfortable	1	2	0	0
Transportation, n				
Not at all	2	0	4	2
Somewhat	5	5	6	6
Frequently/comfortable or very frequently/comfortable	3	5	0	2
Supplemental income, n				
Not at all	10	1	10	5
Somewhat	0	0	0	5
Frequently/comfortable or very frequently/comfortable	0	0	0	0

**Table 2:** Total knowledge score and percent correct responses for each item pre- and post-training (*N* = 10).

	Pre	Post
Total score (potential range 0–6), *mean* (SD)	2.2 (1.4)	3.9 (1.2)
Which of the following are typically included in an out-of-pocket maximum?, n		
a. Deductible	1	1
b. Co-payments	0	0
c. Co-insurance	0	0
d. Premiums	0	0
**e. a, b, and c**	3	0
f. All of the above	6	9
A patient should pay a medical bill…, n		
a. As soon as they receive it from their healthcare provider	0	0
**b. Not until they receive the explanation of benefits from their insurance company**	5	10
c. Any time before the due date	5	0
d. If their provider is refusing to continue care until the bill is paid	0	0
Medicare Part B will pay 100% of a patient’s bills…, n		
a. Once they have hit their out-of-pocket maximum	3	6
b. Once they have met their deductible	1	1
c. If they have a Medicare supplemental plan	5	2
**d. Never**	1	1
Patients may use their accrued paid time off and sick time concurrently with FMLA…, n		
a. Only if required by their employer	2	1
b. Only if they choose to do so	1	2
**c. Either if required by their employer or if they choose to do so**	6	7
d. Never	1	0
An individual may receive SSDI and collect private long-term disability insurance benefits at the same time., n		
**a. True**	3	5
b. False	7	5
Which of the following statements are true about reasonable accommodations?, n		
a. An employer can deny a reasonable accommodation to someone who has not been employed at least 12 months	0	0
**b. Employers are required to provide a reasonable accommodation to eligible employees unless it is an undue hardship or direct threat**	4	7
c. Eligible employees are entitled to one accommodation per year	0	1
d. None of the above	1	0
e. All of the above	5	2

Note: correct responses are indicated in bold.

**Table 3:** Descriptive summary of thoughts on the training (*N* = 10).

Question	*Mean* (SD)
Did the training you receive offer actionable approach for improving your ability to assist patients and/or caregiver with their financial and/or legal needs	2.8 (0.8)
Did the training improve your ability to assist patients and/pr caregivers with their financial and/or legal needs	2.9 (0.7)
Is the training you received suitable or appropriate for the way that you do/your role?	2.2 (1.0)
Are you now more willing to use the information, resources and training your received to engage in assisting your patients/families with financial and/or legal needs?	2.9 (0.9)
Would you recommend this training to other nurses, healthcare providers and staff who work with patients/caregivers with childhood cancers?	3.1 (0.7)

Note: response options ranged from 0, i.e., ‘not at all’, to 4, i.e., ‘very much’.

## Data Availability

Data from this study are not publicly available to protect the identity of participants but are available from the corresponding author on reasonable request.
